# Olfactory Dysfunction in Patients Infected with 2019 Novel Coronavirus

**DOI:** 10.22038/ijorl.2021.51614.2750

**Published:** 2021-05

**Authors:** Mehdi Bakhshaee, Maral Barzegar-Amini, Zohreh Motedayen, Roshanak Khojasteh-Taheri, Mahdi Rafiee, Mahnaz Amini, Parvaneh Layegh, Kamila Hashemzadeh, Delaram Omidvar, Peter H. Hwang, Masoomeh Hosseinpoor

**Affiliations:** 1 *Sinus and Surgical Endoscopic Research Center, Mashhad University of Medical Sciences, Mashhad, Iran. *; 2 *Allergy Research Center, Mashhad University of Medical Sciences, Mashhad, Iran. *; 3 *Department of Otolaryngology, Mashhad University of Medical Sciences, Mashhad, Iran. *; 4 *Department of Microbiology, Faculty of Science, Neyshaboor Branch, Islamic Azad University, Neyshaboor, Iran. *; 5 *Department of Research, Faculty of Medicine, Mashhad University of Medical Sciences, Mashhad, Iran. *; 6 *Student Research Committee, Faculty of Medicine, Mashhad University of Medical Sciences, Mashhad, Iran. *; 7 *Lung Disease Research Center, Mashhad University of Medical Sciences, Mashhad, Iran.*; 8 *Department of Radiology, School of Medicine, Mashhad University of Medical Sciences, Mashhad, Iran. *; 9 *Rheumatic Disease Research Center, Mashhad University of Medical Sciences, Mashhad, Iran.*; 10 *Department of Internal Medicine, Mashhad University of Medical Sciences, Mashhad, Iran. *; 11 *Department of Rhinology & Endoscopic Skull Base Surgery, Stanford University School of Medicine, Palo Alto, California. *

**Keywords:** COVID-19, Olfactory dysfunction, Smell disorder

## Abstract

**Introduction:**

The current study aimed at investigating the occurrence and features of olfactory dysfunction in patients with confirmed coronavirus disease 2019 (COVID-19) infection.

**Materials and Methods:**

Patients with laboratory and clinically confirmed COVID-19 infection were enrolled in this longitudinal study. They were managed in either the inpatient or outpatient setting. The demographic, clinical, and outcome data were retrieved from patients’ medical records. Olfactory dysfunction features, including the onset pattern, duration, and recovery time were investigated. The visual analog scale (VAS) was utilized as a self-rating subjective measurement of olfactory function.

**Results:**

According to the results, the mean age of the patients (n=502) was obtained at 46.8±18.5 years; moreover, 52.4% and 47.6% of cases were female and male, respectively. It was also revealed that 35.4% and 64.5% of the subjects were outpatients and hospitalized, respectively. Based on the findings, 178 (38.4%) subjects had olfactory dysfunction. The mean values of VAS in hyposmic patients were estimated at 2.5±2.5, 8.3 ±2.1, and 9.4±1.6 at the first evaluation, in 2 weeks, and after 1 month of follow-up (P<0.001). The onset of olfactory dysfunction was more suddenly (58.7%). The majority of cases experienced olfactory dysfunction at the same time as other symptoms 72(51.1%). Based on the results, 0.4% of subjects infected with COVID-19 had olfactory dysfunction as an isolated symptom. The olfactory dysfunction was recovered after 2 weeks in 18 (25.3%) anosmic and 37(46.8%) hyposmic patients.

**Conclusion:**

Olfactory dysfunction seemed to be an important symptom of COVID-19 infection. The occurrence of this disturbance as a transient self-limited condition was significantly higher among female subjects.

## Introduction

The olfactory system or the sense of smell can detect odorants in the air and give an individual a better chance of escaping or avoiding dangerous situations (e.g., spoiled foods, fire, and leaking natural gas) (1,2). Despite numerous functions of the olfactory system, it is regarded as less important, compared to vision or auditory systems (1). Olfactory dysfunction is a common manifestation of otolaryngology outpatient service largely affected by three causes, including upper respiratory tract infection (URTI), nose and sinus diseases, and head injuries. In particular, the occurrence rate of secondary olfactory dysfunction after URTI is estimated at 37.9% (3). In December 2019, a cluster of acute respiratory illnesses, now identified as a novel coronavirus-infected pneumonia (NCIP), occurred in Wuhan, Hubei Province, China(4-9). The most prevalent clinical presentations of this disease include fever, fatigue, dry cough, dyspnea, myalgia, normal or decreased leukocyte counts, and the radiographic sign of pneumonia. In severe cases, there is a probability of the occurrences of organ dysfunction (including shock, acute respiratory distress syndrome [ARDS], acute cardiac injury, and acute kidney injury), septic shock, and death (10). In a case report in 2006, persistent long-term anosmia was reported after recovery from respiratory distress in a patient with the severe acute respiratory syndrome (11). There are some reports on olfactory disturbance during coronavirus disease 2019 (COVID-19) infection (12); nevertheless, the quality and quantity of these symptoms, as well as its relation to other symptoms and signs, complications, and severity of the disease are not well described yet. 

## Material and Methods

Study design and participants

This longitudinal study was conducted on 502 patients referred to COVID-19 referral hospitals, Mashhad University of Medical Sciences, Mashhad, Iran, within March-April 2020. They were diagnosed with COVID-19 infection according to the World Health Organization (WHO) interim guidance and were managed in either the inpatient or outpatient setting (25). The eligible patients to be entered into this research were those with laboratory and clinically confirmed COVID-19 infection based on the last updates of the WHO Guideline of Evaluation and Laboratory Testing for COVID-19. On the other hand, the exclusion criteria were 1) olfactory or gustatory dysfunctions before the pandemic, 2) a history of head trauma, 3) a history of nasal surgery, 4) a history of allergic rhinitis, 5) pregnant women, 6) children under 14 years old, and 7) patients without a laboratory and clinical confirmed COVID-19 infection (13).

Data collection 

Demographic, clinical, laboratory, radiologic findings, and outcome data, were collected from patients’ medical records. Clinical outcomes were followed up until April 20, 2020. In case that data were missed from the records or clarification was needed, they were obtained by direct communication with or calling attending doctors and other healthcare providers. The diagnosis of COVID-19 infection in patients admitted to the hospital was confirmed by real-time reverse transcription-polymerase chain reaction (RT-PCR) using the standard protocol (28), which was provided by Mashhad University of Medical Sciences. In addition, other outpatients were diagnosed by clinical and laboratory tests, including the peripheral blood lymphocyte and C-reactive protein (CRP) concentration (10,13). 

Olfactory assessment

All cases were examined by direct communication for the first time and followed up for at least 1 month, prospectively. Olfactory dysfunction features investigated in this study included the onset pattern of anosmia, hyposmia, and parosmia, as well as the duration of olfactory dysfunction and recovery time. Olfactory function in patients was evaluated based on the patient's self-assessment using a single question, such as “How would you estimate your sense of smell?” All patients' replies on their sense of smell were rated on a 10-point visual analog scale (VAS) (0=anosmia; 10=normal Sense of smell). Patients were followed up via phone to assess olfactory function, on the 1^st^, 7^th^, 14^th^, and 30^th^ days after treatment. 

Clinical assessment

All computed-tomography (CT) images were reviewed by a radiology expert for the presence of ground-glass opacity, consolidation, reticular pattern, honeycomb pattern, and mixed pattern. The CT images were evaluated using a method published previously (14). In brief, each lung was divided into 3 zones, and each zone was assessed for the percentage of lung involvement on a scale of 0-4 (0%-100%). The overall CT score was obtained at 24 (14). The complications of the disease were diagnosed in the follow-up. Acute respiratory distress syndrome was defined according to the Berlin definition (15). Acute kidney injury was recognized according to Kidney Disease Improving Global Outcomes (16). The cardiac injury was described if the serum levels of cardiac biomarkers (e.g., troponin I) were above the 99^th^ percentile upper reference limit, or new abnormalities were demonstrated in electrocardiography and echocardiography (10). All data were checked by two physicians.

Ethical considerations

The study was approved by the Ethical Committee of Mashhad University of Medical Sciences (Code: IR.MUMS.REC.1399.131). Written and verbal informed consent were obtained from patients before the enrolment when data were collected retrospectively. 

Data analysis

The obtained data were analyzed in SPSS software (version 22.0; IBM Corp, Armonk, NY, USA). The Kolmogorov-Smirnov test was performed to assess normal distribution. The normal and abnormal quantitative data were respectively expressed as mean±standard deviation (SD) by the one-sample t-test and median± interquartile range by the Mann-Whitney test. The Chi-square test was performed for qualitative data and expressed as number (percentage). A p-value of less than 0.05 was considered statistically significant.

## Results

Patients who managed in outpatient and inpatient settings with confirmed NCIP were enrolled in this cross-sectional study. Baseline demographic characteristics are presented in Table1.

**Table1:** Baseline demographic and clinical characteristics of 2019-novel-coronavirus-infected patients

**Variable**	**Olfactory-dysfunction**	**Non olfactory-dysfunction**	**P-value**
Age (years old)	45.50 ±15.63	47.20±19.32	<0.001
Geder:			
Male	86 (49.4%)	126 (45.5%)	0.421
Female	88 (50.6%)	153 (54.4%)	0.421
Addiction:			
Drugs	8 (4.88%)	15 (6.27%)	0.552
Smoking	11 (6.40%)	22 (8.60%)	0.413
Hookah	6 (3.46%)	9 (3.55%)	0.961
Condition:			
Outpatient	67 (38.72%)	106 (61.27%)	0.921
Inpatient	108 (38.16%)	175 (61.83%)	0.921
Total	178 (38.4%)	286 (61.8%)	

**Fig 1:**
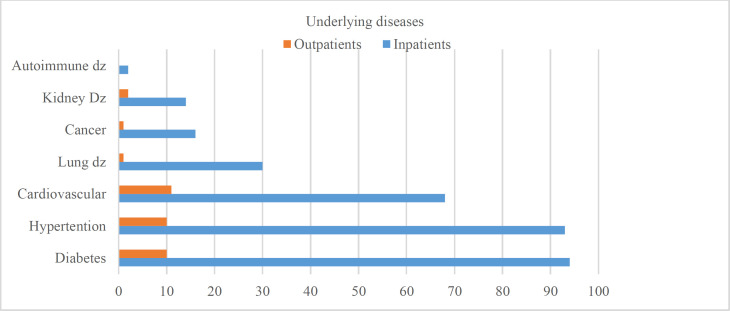
The frequency of underlying diseases in COVID-19 infected patients (inpatients and outpatients)

In this study, out of 502 participants, 178 (35.5%) and 324 (64.5%) of cases were outpatients and hospitalized, respectively. The mean age of patients was reported as 46.8±18.5 years (16-95 years old), and approximately 47.6% of subjects were male. The mean age scores of outpatient and inpatient participants were obtained at 33.1±14.2 and 53.7±16.5 years, respectively. The outpatients were significantly younger than hospitalized patients (P<0.001). Considering the risk factors, hospitalized patients had more underlying diseases, including diabetes, hypertension, and cardiovascular diseases (P<0.001). The frequency of underlying diseases related to both inpatients and outpatients is displayed in Figure 1.

Olfactory outcomes

It was revealed that 178 (38.4%) of subjects with the mean age of 45.5±15.6 years had olfactory dysfunction, while the rest of the cases with the mean age of 47.2±19.3 years lacked olfactory dysfunction. 

There was a significant relationship between age and olfactory dysfunction (P<0.001); therefore, younger patients had a higher incidence of olfactory disturbance. The incidence rates of olfactory dysfunction were estimated at 38.7% and 37.7% in outpatient and hospitalized patients, respectively. Although the incidence rate of olfactory dysfunction was higher in the outpatient participants, no significant relationship was observed between olfactory dysfunction and the condition of patients (P=0.904). Furthermore, the relationship between the severity of hyposmia and the condition of patients in the first examination was assessed. It was also found that there was a notable association between inpatients and outpatients (1.50 vs 3.64; P<0.001). Olfactory features between the two groups of inpatients and outpatients are tabulated in Table 2.

**Table 2:** Olfactory dysfunction features between the two groups of inpatients and outpatients

**Variable**		**Outpatients**	**Inpatients**	**Total**	**P-value**
Olfactory dysfunction		67 (38.3%)	106 (37.7%)	173 (37.9%)	0.904
Anosmia		46 (25.8%)	62 (19.8%)	108 (22.0%)	0.121
Hyposmia		45 (25.4%)	49 (15.6%)	94 (19.1%)	0.008*
Parasmia		6 (3.4%)	11 (3.9%)	17 (3.7%)	0.772
Increasing of smell		3 (1.8%)	2 (0.7%)	5 (1.1%)	0.287
Dysgeusia		34 (19.2%)	64 (22.2%)	98 (21.1%)	0.439
Hyposmia Pattern	Sudden	44 (73.3%)	27 (46.6%)	71 (60.2%)	0.003*
	Gradual	16 (26.7%)	31 (53.4%)	47 (39.8%)	
Anosmia status	Permanent	33 (55.9%)	20 (37.0%)	53 (46.9%)	0.044*
	Transient	26 (44.1%)	34 (63.0%)	60 (53.1%)	
Hyposmia starting time	After recovery	12 (20.0%)	39 (51.3%)	51 (37.5%)	0.001*
	During illness	38 (63.3%)	30 (39.5%)	68 (50.0%)	
	Before symptoms	10 (16.7%)	7 (9.2%)	17 (12.5%)	

The percentage of different types of olfactory dysfunction were obtained at 22.1%, 19.1%, 3.8%, and 1%to for anosmia, hyposmia, parosmia, and increased smell sense, respectively. Moreover, dysgeusia was observed in 11.2% of patients, and 6 patients had hyposmia and parosmia concurrently. It was also revealed that 38% and 62% of the anosmic patients were male and female, respectively. 

The frequency of hyposmia was estimated at 52 (54.7%) and 43 (45.3%) among men and women subjects, respectively. In addition, the prevalence of parosmia was calculated at 9 (47.4%) in males and 10 (52.6%) in females. There was a significant relationship between anosmia and gender (P=0.023); nevertheless, gender was not significantly correlated with hyposmia and parosmia (P=0.120 and P=0.985, respectively). 

The mean of VAS in hyposmic patients was obtained at 4.7±3.7 at the first evaluation (3.2±2.8 in outpatients and it was 8.1±3.3 in inpatients which was 2.5±2.5 at the first evaluation, 8.3 ±2.1 in 2 weeks follow-up, and 9.4±1.6 after 1 month of follow-up. According to the results of paired sample t-test, the mean of VAS was significant between the baseline and 2 weeks, also, there was significant association between the mean of VAS in 2 weeks and a month follow-up, and baseline and a month follow-up (P<0.001). 

The onset of olfactory dysfunction was gradual and sudden in 50 (41.3%) and 71 (58.7%) cases, respectively. The majority of subjects experienced olfactory dysfunction simultaneously with other symptoms 72 (51.1%). Moreover, 17 (12.1%) patients experienced olfactory dysfunction as a presenting symptom before other symptoms, and 52 (36.9%) patients complained about olfactory dysfunction after other COVID-19 symptoms and during disease presentation. In addition, 0.4% of all subjects infected with COVID-19 had olfactory dysfunction as the only symptom, who were females.

**Fig 2:**
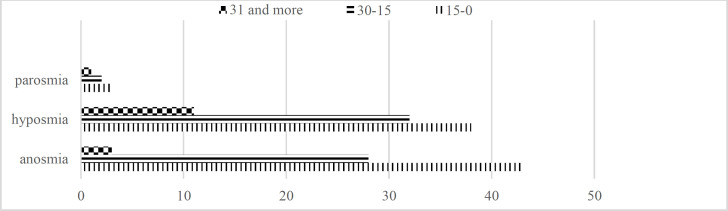
Olfactory dysfunction improvement in one month follow up

The mean time scores of anosmia, hyposmia, and parosmia in subjects were calculated at 16.8±9.6 (4-50), 20.7±15.8 (4-90), and 24.7±17.4 (10-50) days, respectively. All subjects diagnosed with olfactory dysfunction were followed up for a month. Among patients diagnosed with anosmia, 18 (25.3%) cases recovered after 15 days, 24 (33.8%) subjects improved and became hyposmic after 2 weeks, 17 (23.9%) patients recovered after 1 month, 9 (12.6%) cases improved and became hyposmic after 1 month, while 3 (4.2%) anomic patients did not recover until follow-up. Moreover, 37(46.8%) hyposmic patients recovered after 15 days, 31 (39.2%) cases recovered after 1 month, and 11 (13.9%) subjects did not recover until follow-up; however, they showed some improvement during follow-up. Among patients with parosmia, 3 (30%) and 6 (60%) cases recovered after respectively 15 days and 1 month, whereas 1 (10%) patient did not recover until follow up (Figure2). The relationship between olfactory dysfunction and other symptoms was also analyzed (Table.3).

**Table 3:** Relationship between olfactory dysfunction and symptoms

**Symptom**	**Olf-dys**	**Non-olf-dys**	**P-value**
Headache	123 (69.1%)	92 (33%)	<0.001
Fever	116 (66%)	123 (43.6%)	<0.001
Sore throat	47 (27%)	26 (10%)	<0.001
Rhinorrhea	29 (16.5%)	21 (7.8%)	0.004
Nasal congestion	39 (22.1%)	27 (10%)	<0.001
Cough	151 (86.8%)	134 (47.3%)	<0.001
Sneeze	31 (17.6%)	20 (7.4%)	0.001
Dysgeusia	84 (48%)	16 (6%)	<0.001
Dyspnea	127 (71.3%)	138 (48.8%)	<0.001
Eye redness	16 (9%)	6 (2.1%)	0.001

Olf-dys: Olfactory-dysfunction

Based on Table 3, there was a significant relationship between olfactory dysfunction and nasal congestion (P<0.001). The results of multivariate analysis showed that nasal congestion had a positive correlation with olfactory dysfunction (OR=0.451, 95% confidence interval: 0.222-0.917, P=0.028).

Clinical outcomes

**Fig 3:**
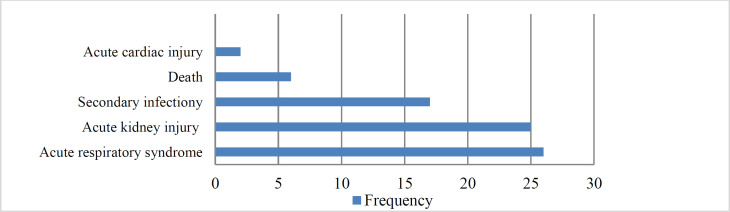
The complication of COVID-19 infected patients

The frequency of complications is depicted in Figure 3. 

 The occurrence of complications was higher in patients without olfactory dysfunction (38 vs. 25); nonetheless, no statistically significant changes were observed (P=0.659). The mean severity scores of radiology findings in CT images were obtained at 11.6±5.9 and 9.7±6.2 in patients with olfactory dysfunction and without complaint of olfactory dysfunction, respectively; however, the difference was not significantly different (P=0.786).

The mean concentration of CRP was reported as 68.6±69.1 and 82.8±126.7 in patients with and without olfactory dysfunction, respectively. No significant relationship was observed between olfactory dysfunction and CRP concentration (P=0.951). 

The mean lymphocyte count in patients with olfactory dysfunction was estimated at 920.2±688.0, while it was calculated at 866.7±625.9 in cases without olfactory dysfunction. There was no significant relationship between olfactory dysfunction and lymphocyte count (P=0.125).

## Discussion

Considering the different recently described symptoms of COVID-19 infection, the detection of unusual manifestations is indispensable for early diagnosis and prevention of infection. Regarding this, the current study assessed the prevalence and follow-up of olfactory dysfunction among COVID-19 patients.

In some case reports, olfactory dysfunction was reported as a symptom among COVID-19 infected patients (11,17-19). Based on the results of an international study conducted in Italy, Spain, the UK, the USA, Germany, France, Iran, and the Netherlands in 2020, Google trends were used to explore internet activity related to loss of smell. 

Accordingly, a strong correlation was revealed between the frequency of searches for smell-related information and the onset of COVID-19 infection in the mentioned countries. They suggested that this may be related to a previously under-recognized symptom (20). Moreover, it was concluded that olfactory dysfunction could be a symptom in COVID-19 infected patients. Based on the obtained results, 38.4% of the subjects complained of olfactory dysfunction. This condition was more evident in younger females. This finding is consistent with that of some studies, such as a recent Iranian study conducted on 10,069 voluntary cases reporting the 48.3% prevalence of olfactory dysfunction. Furthermore, the findings of a case-control study performed on 79 confirmed COVID-19 patients in Spain reported 25 (31.6%) cases of olfactory dysfunction (21,22).

Moreover, in an Italian study carried out on 59 patients, the prevalence of olfactory dysfunction was measured at 14 (23.7%) (23). In another study conducted on a larger sample size of 114 confirmed COVID-19 patients, 54 (47.4%) cases complained of olfactory dysfunction (24). Nonetheless, in a multicenter European study performed on 417 mild-to-moderate COVID-19 patients, 85.6% and 88.0% of patients reported olfactory and gustatory dysfunctions, respectively (25). In addition, in some other studies, the prevalence of olfactory dysfunction was reported higher, compared to that in the current study (26). 

In a case-control study carried out on 68 confirmed COVID-19 patients in France, 51 (75.0%) subjects experienced olfactory dysfunction (27). Moreover, in a larger study performed on 237 subjects in the USA, Italy, the UK, and Mexico, the prevalence of olfactory dysfunction was reported to be 357 (85.6%). Furthermore, the results of a recent Iranian study conducted on 60 confirmed COVID-19 patients reported the 98.3% olfactory dysfunction percentage (26). Based on the findings of two studies performed in China and Italy with respectively a sample size of 214 and 320 COVID-19 patients, the prevalence rates of olfactory dysfunction were obtained at 11 (5.1%) and 62 (19.4%), respectively (19,28). These discrepancies in the results can be attributed to inherent differences of patient populations, regarding both disease severity and setting. In four studies, the inclusion criterion was merely considered to be an inpatient (moderate to severe forms) (23,26,29). However, two other studies involved both inpatients and outpatients (mild to moderate forms) (24, 30), and two studies included outpatients (mild forms) (31,32). Moreover, a wide range of instruments was used to detect olfactory dysfunction, including verbal interviews; non-validated questionnaires; validated surveys; and validated objective testing, such as the University of Pennsylvania Smell Identification Test, which could explain the differences in the prevalence of olfactory dysfunction. In line with the results of the present study, in all of the above-mentioned studies, olfactory dysfunction was higher among females. Non-smokers appear to be much more susceptible than smokers to olfactory dysfunction caused by industrial exposures to acrylate and methacrylate (33), and it seems that smoking protects, to some extent, against the olfactory loss of Parkinson’s disease (34). The lower prevalence of female smokers could explain the higher prevalence of olfactory dysfunction in females. In addition, in most studies, it was found that the severity of COVID-19 was lower in females, and the incidence rate of olfactory dysfunction was higher in outpatients; therefore, this could contribute to the higher frequency of olfactory dysfunction in females. During follow-up, 59.2%, 46.8%, and 25% of anosmic, hyposmic, and parosmic patients had an early recovery (14 days). Bertran-cobellini et al. reported that 40% of patients had complete recovery after 7 days (29). The prevalence of early recovery (10 days) was reported in 85% of subjects in a study conducted by Kaye et al. (32). The early olfactory recovery rate was obtained at 44.0% in a European study (30). Yan et al. reported that 73.7% of patients recovered before 4 weeks (31). Consequently, it seems that olfactory dysfunction is a self-limited disorder and transient condition that quickly improves after the disease subsides. Based on our result, the mean age was lower in participants with olfactory dysfunction than in patients without olfactory dysfunction (45.5±15.6 vs. 47.2±19.3). To the best of our knowledge, no specific study has compared the mean age of subjects having olfactory dysfunction with that of patients lacking olfactory dysfunction; as a result, it was not possible to compare this finding with that of other studies. Concerning the higher incidence rate of olfactory dysfunction in outpatient participants that are younger than hospitalized patients, the lower age range in olfactory dysfunction would be reasonable. Since complication, mortality, and severity of disease were not related to the olfactory disturbance, it seems that the aforementioned disturbance is not a prognostic value for COVID-19. The focus has recently shifted towards the relationship between COVID-19-mediated olfactory dysfunction and other sinonasal symptoms, including nasal congestion and rhinorrhea. In the present study, the frequency of nasal congestion and rhinorrhea in patients with olfactory dysfunction was reported to be 22.1% and 16.5%, respectively. In a similar vein, Leichen et al. reported that 79.7% of COVID-19 patients had anosmia without nasal congestion (30). Moreover, the prevalence of nasal congestion was low in other studies (29,32). This reveals that the olfactory dysfunction may not be related to nasal inflammation and obstruction and can be attributed to direct olfactory system invasion. In the current study, 17 (12.1%) and 72 (51.1%) patients experienced olfactory dysfunction before and simultaneously with other symptoms, respectively. On the contrary, the results of a study carried out by Giacomelli et al. in Italy revealed that 12 (20.3%) 8 (13.5%) patients presented the symptoms before and during hospitalization, respectively. It was also reported that taste changes were more frequent (91%) before hospitalization, whereas after hospitalization, taste and olfactory alteration appeared with equal frequency (30). 

Additionally, anosmia was noted in 73% of patients prior to diagnosis and was the initial symptom in more than a quarter of cases in a study performed by Kaye et al. (32). In a retrospective observational study conducted by Klopfenstein et al., anosmia was never the first or second symptom to develop, rather it was the third symptom in 38% of patients, and anosmia developed 4 days after infection onset (31). Olfactory dysfunction appeared before other symptoms in 11.8% of cases in a study carried out by Lechein et al. (30). Similar to the prior investigation (33), the onset of olfactory dysfunction was more sudden in our subjects.

The remarkable limitations of the current study included the impossibility to perform RT-PCR for all patients; therefore, clinical and laboratory testing were used to confirm infection for outpatients. Furthermore, some patients in a life-threatening condition, such as those who were transferred to the intensive care unit, limited the follow-up procedure; therefore, these cases were excluded. Another limitation was related to olfactory assessment. Although the objective tests may be more reliable than subjective ones for the assessment of hyposmia, it was reasonable to ignore using objective tests to avoid the accumulation of patients and the transmission of coronavirus. 

## Conclusion

There is accumulating anecdotal evidence that olfactory dysfunction is associated with the COVID-19 pandemic. There is a possibility of the presentation of smell loss among such patients, which can be even the sole symptom of this disease in a few patients. This symptom was found to be significantly higher among female subjects. This disturbance is a transient self-limited condition in the majority of cases with sudden occurrence. Although local inflammation seems to be the related reason for this disturbance, further studies are required to be performed to determine the exact mechanism.
